# A rare case of avascular necrosis in sickle cell trait: a case report

**DOI:** 10.1186/s12878-018-0098-z

**Published:** 2018-02-22

**Authors:** William J. Sanders

**Affiliations:** 1 Georgia Campus-Philadelphia College of Osteopathic Medicine (GA-PCOM), Suwanee, GA 30024 USA; 2 Houston Medical Center, Warner Robins, GA 31088 USA

**Keywords:** Sickle cell trait, Avascular necrosis, Sickle cell disease, Total hip arthroplasty, Hip

## Abstract

**Background:**

Sickle cell trait is usually an asymptomatic presentation of a patient with slightly different hemoglobin molecule makeup than normal. It is similar to a more serious disease, sickle cell disease, in which a person’s hemoglobin is mutated in such a way that causes their red blood cells to easily change shape in certain environmental and internal states; this causes red blood cells to adhere to the walls and occlude the lumen of the arteries in which they travel, leading to downstream effects secondary to ischemia. Sickle cell trait does not have these ischemic effects, usually.

**Case presentation:**

In this case, a young African American female patient presents to the clinic with severe right hip pain. Her past medical history includes sickle cell trait and asthma. She has not been symptomatic of her asthma for years and is not on therapy for it. The pain has lasted for several months and has not improved with anti-inflammatory medication. There is severe pain with internal and external rotation of the hip. The neurovascularity of the lower extremities is intact bilaterally. MRI of the femur shows stage 2 or 3 avascular necrosis of the femoral head, while X-rays of the femur are unremarkable. Non weight-bearing for several weeks was unsuccessful; shortly thereafter, the patient underwent core decompression of the right femoral head as well as starting bisphosphonates. The patient improved temporarily but regressed shortly thereafter. Her avascular necrosis worsened radiographically over the next several months. At this point, the only other option would be to do a total hip arthroplasty, but the patient may need several more throughout her lifetime due to the lifespan of the artificial replacement.

**Conclusion:**

There have only been scarce reports of avascular necrosis in patients with sickle cell trait. This manuscript presents such a case and includes the trials and tribulations associated with its management.

## Background

Avascular necrosis (AVN), also known as osteonecrosis, is a condition in which ischemia of bone tissue has taken place, leading to infarction. This can result from trauma such as femoral neck fracture, where there are watershed areas that supply the femoral head that do not cover enough area to allow for compromised blood flow to occur without ischemic consequences. Once infarction takes place, it is only a matter of time before oxidative phosphorylation cannot take place and necrosis ensues. Most living tissues need oxygen, without which there is inefficient metabolic functioning. The prevalence of AVN is around 20,000–30.000 new diagnoses per year. There are a number of atraumatic associated etiologies, such as systemic lupus erythematosus (SLE), radiation therapy, coagulopathy such as factor V leiden mutation, excessive alcohol and glucocorticoid use, and Sickle Cell anemia [1–3]. AVN is multifactorial but can begin with interruption of blood and oxygen supply to vasculature in and around bone and progresses to trabecular thinning (also seen in cases of osteoporosis) and, eventually, collapse of bone. In the case of sickle cell disease, this infarction results from occlusion of the vasculature by red blood cells (RBC’s) which have changed form to flow less smoothly in the blood vessels. The RBC’s take on the shape similar to a crescent and are not as round and freely passible as normal RBC’s. Their shape allows them to adhere to other RBC’s as well as the endothelial walls, worsening vaso-occlusion. This can lead to occlusion of bone marrow, ischemia, and eventual progression to AVN. It is fairly common in sickle cell disease. As much as 50% of sickle cell patients can develop AVN by the time they reach the age of 35. However, it is very rare in sickle cell trait (SCT), a much milder form of sickle cell disease in which patients are usually asymptomatic. This case report intends to shed light on a rarely reported instance of AVN in sickle cell trait and the dilemma encountered during its treatment in a young African American female.

## Case presentation

**Fig. 1:**
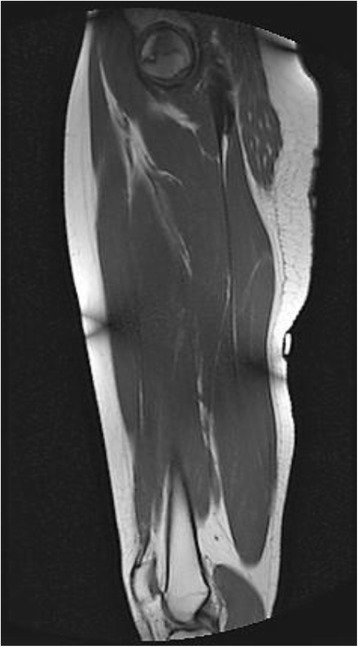
This T1-weighted image shows a serpiginous band of hypodensity that represents the separation point between necrotic bone and bone that is undergoing repair. This is pathognomonic of AVN

The patient is a 22 year old African American female with a past medical history of sickle cell trait and asthma who presents to the orthopedic clinic with aching right hip pain. She-like her father- has sickle cell trait. Throughout her life so far, she has not had any acute episode of sickle cell-related symptoms like severe, acute chest pain. However, she states about a year prior, she had the same hip pain. She went to her primary care provider, who gave her hip cortisone injections. These helped for a few weeks. She then went on vacation a month later. Her feet became swollen during this time, prompting her to visit her primary doctor again. At this time, she was referred to the orthopedic clinic. She had severe groin pain with internal and external rotation of her hip. X-rays were done of her hip, which showed an area of necrosis suggestive of stage 1 or 2 AVN in the head of the right femur. An MRI was done and showed stage 2 or 3 AVN. This can be seen in Fig. 1.

**Fig. 2:**
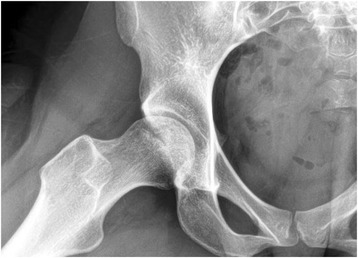
This AP x-ray was done 2 weeks post decompression. The patient was asymptomatic at this point, and x-ray shows a normal femoral head

She was then prescribed bisphosphonates, told to walk with no weight bearing on the right leg, and scheduled for a decompression procedure several weeks later. The plan after surgery was to be minimal weight bearing for 6 weeks as well as continuing therapy with Alendronate, a bisphosphonate. After decompression, the patient’s pain subsided. Two weeks after surgery, the patient presented for follow-up and suture removal. She was in high spirits as her pain and overall anxiety had subsided. She had not felt pain free in quite some time. Her hip x rays were normal at this time. Figure 2 shows x- rays at two weeks post op. At six weeks post-op, she was feeling like she could bear weight on the hip. There was no pain provoked on range of motion testing or internal and external rotation. She was then scheduled to begin physical therapy soon thereafter.

**Fig. 3:**
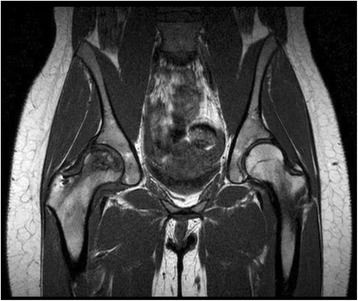
This coronal T1-weighted image shows progression of AVN with necrosis, flattening of the right femoral head, and post decompression evidence in the femoral neck. There is also edema, which corresponds to the increasing pain the patient was experiencing at this time. There are obvious differences between the left and right femoral heads

**Fig. 4:**
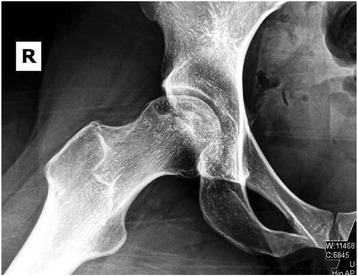
AP view of the right femoral head shows areas of hyperlucency and surrounding sclerosis, as well subtle changes in the shape of the articular surface. The necrosis also spreads into the acetabulum. This x-ray was done just before the patient was referred to a hip revision specialist

However, at eight weeks post op, she presented to the clinic for follow-up stating new onset pain. She had weight-bearing but the pain had started again in her hip and groin. There were also new x-ray findings (AP Pelvis and Frog Hip views), which showed a serpiginous line which was consistent with progression of her AVN with no collapse of the femoral head. Even after bisphosphonates and decompression, the patient’s AVN had now grown to involve the majority of the head of the femur. At 3 months status post- surgery, MRI showed that the head of the femur was beginning to flatten as well as loss of volume and bone marrow edema, which is seen in Fig. 3. Further progression of her AVN can be seen with follow up x-rays at her later appointment 5 months post decompression in Fig. 4.

At this point, there were no other conservative options available for this patient. The dilemma is her age. Osteotomy is sometimes a treatment for AVN, but given the area of necrosis of this patient’s femoral head, this procedure is not indicated. The plan for this patient is to continue non- weight- bearing. The only definitive treatment for this patient would be a total hip arthroplasty (THA). Because this patient is so young, the patient was referred to a hip joint revision specialist with plan to follow-up again 6 weeks later. At her age, it would be challenging to do a THA, given that she would most likely have to undergo the same procedure several more times throughout her life. Hip replacements have an average life of 15–20 years, so the prospect of conserving as much of the joint as possible and not undergoing THA at such a young age is paramount. It would be devastating for the patient to have to have 4–6 hip replacements in her lifetime. Time will tell if conservative therapy such as minimal weight-bearing, bisphosphonates, and vitamin D will have any appreciable effects on this patient’s hip. The progression from seemingly successful surgery - clinically and radiographically - to the onset of pain spanned two months. Everything will be done to buy her time and avoid THA.

## Discussion and conclusions

The normal adult hemoglobin has two alpha and two beta chains. Hemoglobin that has the ability to sickle is referred to as sickle hemoglobin (HbS). Sickle hemoglobin is the result of a single point mutation from Glutamine to Valine on the beta globin chain. If an individual is homozygous-meaning they inherit two mutated beta globin chains- they will have sickle cell disease [4]. These abnormal beta chains cause RBC’s to become fragile and change shape secondary to conditions of hypoxia, acidity, and dehydration. Repeated vaso-occlusion by sickled RBC’s will eventually cause ischemia, infarction, edema, and end-organ damage of bone any system [5, 6].

If an individual is heterozygous, they only inherit one mutated beta globin chain and will have sickle cell trait. It usually takes 50% of HbS for cells to have the ability to sickle. Sickle cell trait individuals have around 40% and therefore are usually asymptomatic [7]. However there are instances of reported exertional rhabdomyolysis, exercise-induced sudden death, renal papillary necrosis, venous thromboembolism (VTE), and fetal demise.

There have only been four to five cases of AVN in sickle cell trait reported in the literature, so it is an extremely rare occurrence [8]. Physicians must be acutely aware of any patients of African descent who present with hip pain. There is always the possibility of the patient having AVN secondary to sickle cell disease. However, there is still theoretically a chance of the patient having AVN even if they only have the trait. We must be ready to have a lower threshold to think about getting conservative treatments as soon as possible, to salvage what we can of the patient’s anatomical, structural integrity of the bone.

More detailed studies need to be undergone to assess the reason why sickle cell trait sometimes has severe exacerbations. There may be an underlying genetic or epigenetic component which allows the RBC’s to undergo change of shape even though on electrophoresis they do not appear to have HbS or HbSC. It will be challenging down the road to assess younger patients who present like the aforementioned one, because their situation exacts special care so that they may not have to undergo numerous hip replacements in their future. The quality of life and economical aspects of this case are very important, so care must be given to treat quickly. Hopefully with newer technology and pharmacologic therapies, along with conservative approaches like non weight-bearing, patients who develop AVN will have a plan in place to salvage bone and quality of life.

## Abbreviations

AVN: Avascular necrosis
SCT: Sickle cell trait
SLE: Systemic lupus erythematosus
